# Cannabinoid Receptors Reduced Early Brain Damage by Regulating NOX‐2 and the NLRP3 Inflammasome in an Animal Model of Intracerebral Hemorrhage

**DOI:** 10.1111/cns.70385

**Published:** 2025-04-17

**Authors:** Ari Misael Martínez‐Torres, Crisalde Ramírez‐Celis, Julio Morán

**Affiliations:** ^1^ División de Neurociencias, Instituto de Fisiología Celular Universidad Nacional Autónoma de México Ciudad de México México

**Keywords:** cannabinoids, CB1 receptor, intracerebral hemorrhage, neuroinflammation, oxidative stress

## Abstract

**Background:**

Intracerebral hemorrhage (ICH) is a leading cause of death and disability worldwide. Following the initial mechanical injury caused by hematoma expansion, a secondary injury occurs, characterized by the production of reactive oxygen species (ROS) generated by NOX‐2 and neuroinflammation, which is exacerbated by the upregulation of the NLRP3 inflammasome. These conditions collectively aggravate brain damage. The endocannabinoid system (ECS), through the activation of the cannabinoid receptors, has demonstrated neuroprotective properties in various models of brain injury. However, the role of the ECS during ICH remains poorly understood, particularly regarding the action of the CB1 receptor in the activation of NOX‐2 and the inflammasome. The present study investigates the neuroprotective effects of the cannabinoid receptor agonist WIN55,212‐2 in an ICH animal model, specifically examining the roles of NLRP3 and NOX‐2.

**Methods:**

Male C57BL/6 mice were subjected to ICH through an intracerebral injection of collagenase, followed by intraperitoneal administration of WIN55,212‐2 and/or MCC950, a selective NLRP3 inhibitor. Various outcome measures were employed, including assessments of motor activity, hematoma volume, brain water content, and blood–brain barrier (BBB) permeability, which was evaluated using Evans blue assay. Additionally, the activity of NOX and the protein levels of crucial markers such as CB1, gp91phox, NLRP3, AQP4, and caspase‐1 were measured via western blot analysis.

**Result:**

The findings demonstrate that ICH induced a significant brain lesion characterized by hematoma formation, edema, BBB disruption, and subsequent motor impairments in the affected mice. Notably, these detrimental effects were markedly reduced in animals treated with WIN55,212‐2. The study also revealed an activation of both NOX‐2 and NLRP3 in response to ICH, which was reduced by cannabinoid receptor activation. Furthermore, the pharmacological inhibition of NLRP3 using MCC950 also led to a reduction in hematoma size, edema, and motor impairment secondary to ICH.

**Conclusions:**

These results support a neuroprotective role of the cannabinoid receptor activation during ICH and suggest the involvement of NOX‐2 and NLRP3.

## Graphical abstract

During intracerebral hemorrhage, reactive oxygen species, produced by NADPH‐oxidase 2 (NOX‐2), are increased and the NLRP3 inflammasome is activated. This increases aquaporin‐4 expression, alters the blood–brain barrier, and produces cerebral edema and neuronal death. Stimulation of CB1R reduces NOX‐2 levels and NLRP3 activation caused by intracerebral hemorrhage, reducing brain injury.

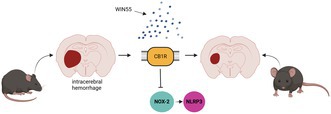

## 1 Introduction

Intracerebral hemorrhage (ICH) represents a significant subtype of hemorrhagic stroke, characterized by elevated mortality rates and long‐term disability among affected individuals [1]. The current therapeutic options to reduce neuronal damage following ICH are notably limited, highlighting the necessity for further investigation into potential neuroprotective targets. The pathophysiological mechanisms contributing to ICH‐related damage can be categorized into two primary categories: primary and secondary injuries [2]. Primary injury is characterized by increased intracranial pressure resulting from hematoma expansion, which leads to the compression of adjacent brain regions, reduced cerebral blood flow, and potential brain herniation. Furthermore, the processes of edema and disruption of the blood–brain barrier (BBB) exacerbate the expansion of the hematoma [3]. In contrast, secondary injury involves the molecular pathways activated in response to the initial hematoma expansion, with a particular emphasis on inflammatory processes. This immediate inflammatory response initiates the activation of microglia. It facilitates the infiltration of immune cells, culminating in the release of pro‐inflammatory cytokines, such as interleukin 1‐β (IL‐1β) and tumor necrosis factor α (TNF‐α). These inflammatory mediators play a critical role in the progression of neuronal damage following ICH. The Nucleotide Oligomerization Domain (NOD)‐like Receptor Protein 3 (NLRP3) inflammasome is a critical component of the innate immune system, exhibiting significant expression levels in microglia and macrophages. This cytosolic multiprotein complex consists of NLRP3, caspase‐1, and the apoptosis‐associated speck‐like protein containing a caspase recruitment domain CARD (ASC). Upon activation, the NLRP3 inflammasome catalyzes the activation of procaspase‐1, leading to the production of cleaved caspase‐1, which subsequently generates the inflammatory cytokines interleukin‐1 beta (IL‐1β) and IL‐18 [4]. NLRP3 has been implicated in a range of acquired and neurodegenerative brain diseases. In experimental models of both hemorrhagic and ischemic stroke, the absence or pharmacological inhibition of NLRP3 has demonstrated a reduction in brain edema, lesion volume, and neurological dysfunction [5, 6, 7, 8, 9, 10, 11]. These findings suggest that targeting the NLRP3 inflammasome may offer therapeutic potential in mitigating the adverse effects associated with these neurological conditions.

Oxidative stress mediates secondary injury and activates the NLRP3 inflammasome during ICH. NADPH oxidases (NOX), particularly NOX‐2, are significant sources of reactive oxygen species (ROS) within the nervous system and have been implicated in neuronal damage after traumatic brain injury (TBI), as well as ischemic and hemorrhagic strokes [12, 13, 14]. During the recovery phase following ICH, an increase in NOX‐2 protein levels and IL‐1β has been observed [15, 16, 17]. In transgenic mouse models subjected to TBI, the absence of NOX‐2 has been shown to reduce the expression of critical components of the inflammasome complex, including NLRP3, ASC, caspase‐1, and IL‐1β [18]. These findings support the dependence of inflammasome activation on NOX‐2 and emphasize the significant role of oxidative stress in neuroinflammation.

The Endocannabinoid System (ECS) is extensively distributed throughout the brain and plays a significant role in neurodevelopment and synaptic plasticity. Furthermore, it has been associated with various neuroprotective mechanisms [19]. The ECS comprises two primary G protein‐coupled receptors (GPCRs): type 1 (CB1) and type 2 (CB2), which are characterized by their seven transmembrane domains. Additionally, the system includes the endocannabinoids arachidonoyl ethanolamide (commonly known as anandamide) and 2‐arachidonoyl glycerol (2‐AG), along with the enzymes responsible for their synthesis and degradation [20].

ECS activation has demonstrated protective effects during brain injury [21]. In the experimental model of subarachnoid hemorrhage (SAH), treatment with JWH133, a CB2 agonist, resulted in reduced neurological impairment and edema [22]. Additionally, in an animal model of ICH, JWH133 facilitated the transition of microglia M2 phenotype, thereby regulating inflammation, neuronal death, and brain edema [23, 24]. ECS has been implicated in modulating oxidative stress during neuronal injury. In a study with quinolinic acid‐induced neurotoxicity in rat striatal primary cell cultures, the CB1 agonist WIN55212‐2 (WIN55) effectively prevented lipid peroxidation and reduced the formation of ROS [25]. Similarly, URB597, an inhibitor of anandamide degradation, enhanced the activities of catalase and SOD, while it reversed the increase of ROS and malondialdehyde (MDA) levels in BMEC cell cultures subjected to oxygen–glucose deprivation (OGD) [26]. Furthermore, WIN55 was shown to decrease the expression of p47phox and Rac‐1, components of the NOX‐2 complex, in a model of neurotoxicity induced by MPTP [27]. The NLRP3 inflammasome plays a significant role in neuroinflammation following ICH, with NOX‐2 identified as a source of ROS that activates the NLRP3 inflammasome. While the ECS is known to exert antioxidant and neuroprotective effects post‐injury; however, the mechanisms responsible for this action, particularly the regulation of ROS levels and the activation of NOX2 and NLRP3 inflammasome, remain unclear. This study aims to elucidate the roles of NOX‐2 and the NLRP3 inflammasome in the neuroprotective effects of WIN55, a CB1 and CB2 agonist, using an in vivo model of ICH. The investigation will encompass assessments of motor activity, lesion volume, NOX activity, brain edema, blood–brain barrier (BBB) disruption, and aquaporin‐4 (AQP4) levels.

## 2 Materials and Methods

### 2.1 Animals

Animals were managed under the National Institutes of Health Guide for the Care and Use of Laboratory Animals (NIH Publication No. 8023, revised 1978) and the Local Committee for the Care and Use of Laboratory Animals (CICUAL protocol JMA222‐23). Efforts were implemented to minimize pain and reduce the number of animals used in the study. Adult male mice C57BL/6 (10 weeks old) were obtained from the bioterium of the Instituto de Fisiología Celular at the Universidad Nacional Autónoma de México. The mice were housed under controlled temperature conditions (20°C–22°C) with a regulated 12‐h light–dark cycle, and they had ad libitum access to food and water.

### 2.2 Intracerebral Hemorrhage Model and Post‐Procedure Treatment

Intracerebral hemorrhage (ICH) was induced via the intracerebral injection of bacterial collagenase [28]. The animals were anesthetized using a combination of ketamine (100 mg/kg) and xylazine (10 mg/kg) and administered intraperitoneally. They were then positioned on a stereotaxic frame where a stainless‐steel needle was inserted into the right striatum, guided by the following coordinates: anteroposterior +0.8 mm from bregma, lateral +1.8 mm from the midline, and vertical 3.2 mm from the dura at the designated site in the right striatum. An injection pump (KDS 210 Kd Scientific) delivered 0.075 U of Collagenase type VII (Sigma‐Aldrich, C‐0773) dissolved in 0.5 μL of a 0.9% saline solution at a rate of 0.1 μL/min [29]. After the injection, the needle was carefully withdrawn, and the incision was sealed with Dermabond ProPen. Post‐procedure, the mice received an intraperitoneal injection of either AM251 (2 mg/kg) or WIN55 (3 mg/kg). A subset of animals was pre‐treated with the NLRP3 inhibitor MCC950 (Sigma‐Aldrich, PZ0280) at a dosage of 50 mg/kg, administered intraperitoneally 1 h prior to surgery, followed by WIN55 administration post‐surgery. Dosages were determined based on prior studies [30, 31, 32]. All drugs were dissolved in dimethyl sulfoxide (DMSO) and subsequently diluted in saline, with SHAM groups receiving the same vehicle treatment. The mice were allowed to recover from anesthesia in a temperature‐controlled chamber and were placed in individual cages with ad libitum access to food and water.

### 2.3 Cylinder Test for Forelimb Asymmetry

The cylinder test was employed to assess unilateral forelimb use in mice following intracerebral hemorrhage [33]. Mice were placed in a glass cylinder (11 cm in diameter) on a transparent tabletop for 3 min, during which the number of unilateral and bilateral wall contacts were recorded. The percentages of bilateral and unilateral contacts were calculated using the formulas: 100 × (bilateral contacts/total forelimb wall contacts) and 100 × (unilateral contacts/total forelimb wall contacts). The results are presented as the percentage of unilateral exploration.

### 2.4 Inverted Grid Test

This study evaluated limb strength and coordination in mice [34]. Mice were positioned at the center of a 20 cm by 20 cm wire mesh grid, featuring openings of 0.5 cm, and enclosed by wooden walls. The grid was elevated 20 cm above a tabletop and data were recorded over a 60 s period. No pretraining was conducted; however, a 30 s acclimation pre‐test was performed before the experimental day.

### 2.5 NOX Activity

NOX activity was assessed in striatal homogenates prepared with lysis buffer 24 h post‐intracerebral hemorrhage. The measurement involved quantifying the oxidation of dihydroethidium (DHE) to ethidium (Et) [14]. Tissue homogenates were incubated with 0.02 mM DHE (Sigma, 37291), 0.5 mg/mL salmon DNA (Behringer, 1146714), and 0.2 mM NADPH (Sigma, N7505) as substrates. Ethidium (Et) fluorescence was recorded over 30 m at an excitation wavelength of 480 nm and an emission of 610 nm using a Synergy HT Multi‐detection microplate reader (Biotek Instruments, Colchester, VT). Samples were prepared in duplicates, and NOX activity was calculated as Et fluorescence per milligram of protein per minute, relative to the SHAM group.

### 2.6 Western Blot

At 24 h post‐ICH, animals were anesthetized and decapitated to dissect the striatum. Tissue samples (15 to 20 mg) were homogenized in RIPA buffer with protease inhibitors at 4°C using an OMNI TC homogenizer and centrifuged at 5000 × *g* for 5 min. Cytosolic and membrane fractions were isolated using the Mem‐PER Plus Membrane Protein Extraction Kit (Thermo Scientific cat# 89842). Protein concentrations were measured with a DC Protein Assay kit (BIO‐RAD, cat# 5000111) following the manufacturer's guidelines. Protein homogenates (50 μg per lane) were loaded onto a 12.5% native gradient gel, subjected to SDS‐PAGE, and transferred to a PVDF membrane using tris‐glycine‐methanol transfer buffer at 100 V for 75 min at 4°C. Membranes were blocked overnight with 5% nonfat dry milk in PBS and incubated with various primary antibodies, including anti‐gp91phox (1/5000; 12906, Abcam, USA), anti‐AQP4 (1/5000, AQP‐004, Alomone Labs, Israel), anti‐CB1 (1/5000, sc‐518035; SCBT, USA), anti‐NLRP3 (1/5000, Novus biologicals, NBP2‐12446), anti‐caspase 1(1/3000, sc‐56036, SCBT, USA) anti‐GAPDH (1/5000, 14C10, Cell signaling, USA), and anti‐Na+/K + ‐ATPase α1 (1/3000, sc‐514614, SCBT, USA) antibodies. After washing, membranes were incubated with an alkaline phosphatase‐conjugated secondary antibody (1/50000) for 1 h. Protein bands were visualized using a C‐DiGit Blot Scanner (LI‐COR, USA). Initially, the blots were probed for GAPDH antibody. Following this, they were stripped and probed with additional primary antibodies. The outcomes were presented as the proportion of the protein of interest to GAPDH.

### 2.7 Brain Water Content

At 24 h post‐ICH, the animals were anesthetized and subsequently decapitated to extract and weigh their brains. Following this, the tissue was dried at 100°C for 24 h, and the dry weight was documented. The percentage of water content in each hemisphere was calculated using the following equation [35]:

$$\begin{array}{cc} \%\text{hemisphere water content}\;= & \left(\mathrm{wet}\;\text{weight}-\mathrm{dry}\;\text{weight}\right)\\ & \times 100/\mathrm{wet}\;\text{weight}. \end{array}$$

The individual percentage of change among hemispheres was calculated utilizing the equation:

$$\begin{array}{cc} \%\text{individual water change content}\;= & \text{ipsilateral}\%\text{water content}\\ & -\text{contralateral}\%\text{water content}. \end{array}$$

### 2.8 Brain–Blood Barrier Permeability

After 24 h of intracerebral injection, mice received an i.p. dose of 4 mL/kg of 2% Evans blue solution (Sigma, E2129). One hour later, the mice were infused with 80 mL of 1X PBS. The brains were extracted, and the right stratum was dissected, weighed, and homogenized in 1 mL of 1X PBS, vortexed, and 1 mL of 60% trichloroacetic acid (Sigma, T915) was added. Samples were stored at 4°C for 30 min, before centrifugation at 16,000 rpm. A 250 μL of the supernatant was placed on a microplate and Evans blue concentration was measured at a wavelength of 610 nm. Results were calculated from a standard curve and expressed in μg per mg of tissue [36].

### 2.9 Hematoma Volume Quantification

The hematoma volume was evaluated 24 h after ICH. Mice were anesthetized with ketamine/xylazine and transcardiacally perfused with 60 mL of 0.9% saline, followed by 40 mL of 4% paraformaldehyde solution in 0.1 mM phosphate buffer (pH 7.4). Brains were removed and fixed in the same solution at 4°C overnight, then dehydrated with 10% sucrose for 24 h and 30% sucrose for 72 h. Consecutive coronal sections (40 μm thick) were obtained in a cryostat (CM1850, Leica, Microsystems Nussloch GmbH, Heidelberg, Germany) and stained with cresyl violet. Sections with lesions were selected for volume measurement. Areas of damage were outlined by hand and quantified using the Image J software program (Image J version 1.48v; Wayne Rasband, National Institutes of Health, USA) by an analyst unaware of the treatment details. The total lesion volume was calculated by summing the volumes of the damaged sections, each multiplied by the slice thickness of 40 μm.

### 2.10 Statistical Analysis

Data are presented as the mean ± standard deviation. Statistical analysis was performed using GraphPad Prism version 8.0, (California, USA). The Shapiro‐Wilks test was used to assess data distribution; normally distributed data underwent analysis of variance with Tukey's post hoc test for multiple comparisons. The data without normal distribution was analyzed with a non‐parametric equivalent Kruskall‐Wallis with further post hoc analysis using Dunn's test for multiple comparisons. A *p*‐value of less than 0.05 (*p* < 0.05) was considered to indicate statistically significant differences.

## 3 Results

### 3.1 CB1 Protein Levels Increase in Response to ICH

**FIGURE 1:**
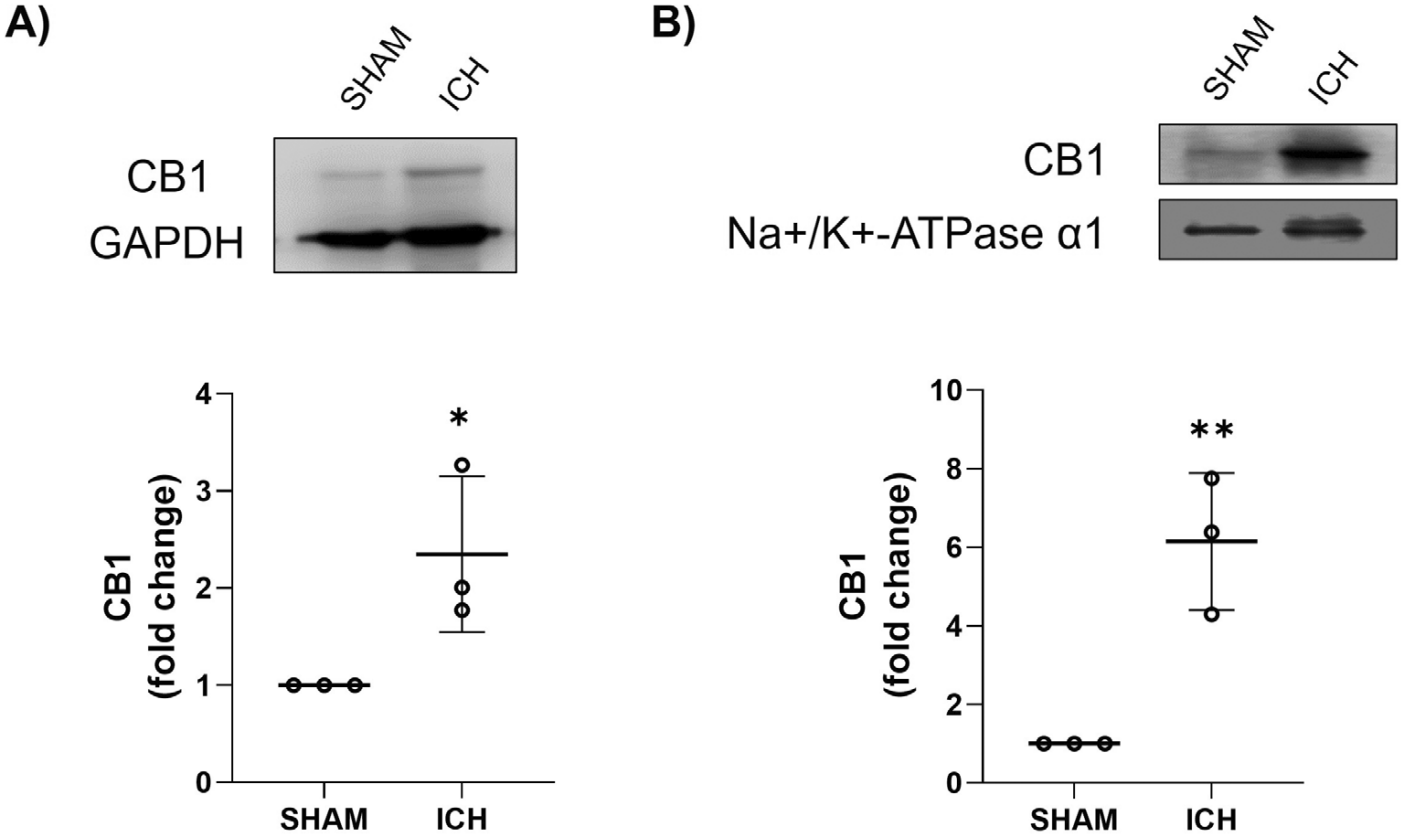
CB1 levels in cytosolic (A) and membranal (B) fractions in animals after the ICH. Western Blot of CB1 and GAPDH representative images (*n* = 3) and levels of CB1. Results are expressed as fold change. Values represent the mean ± standard deviation. GAPDH (37 kDa), CB1 (64 kDa), Na+/K + ‐ATPase α1 (100 kDa). *p < 0.05, **p < 0.01 vs. SHAM group.

We observed that CB1 is present in both the cytosolic (Figure 1A) and membranal fraction (Figure 1B) of the striatum. After 24 h of intracerebral hemorrhage induction, CB1 levels significantly increased in both fractions (Figure 1A,B).

### 3.2 WIN55 Reduces Motor Dysfunction and Hematoma Volume Caused by ICH

**FIGURE 2:**
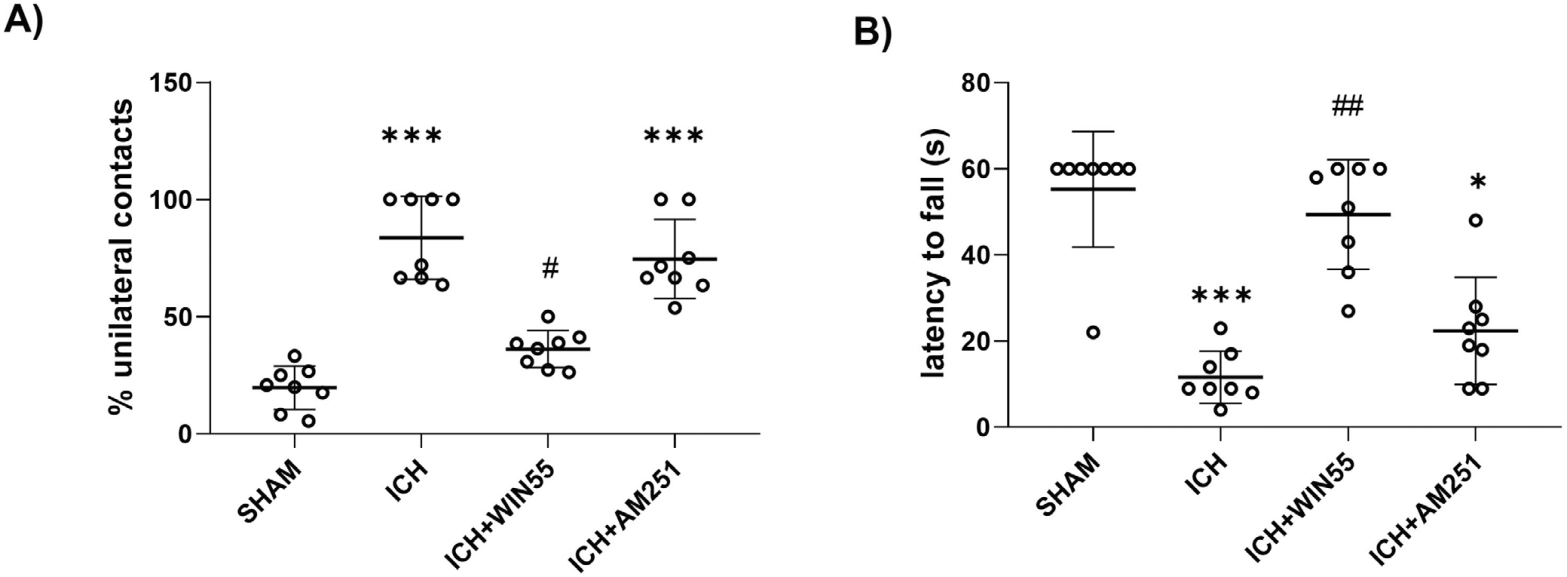
Motor activity recovery in animals after ICH and treated with WIN55 or AM251. (A) Cylinder test. (B) Inverted grid test (*n* = 8). Results were expressed as means ± standard deviation *p < 0.05, ***p < 0.001 vs. the SHAM; # < 0.05, ##*p* < 0.01 vs. the ICH group.

**FIGURE 3:**
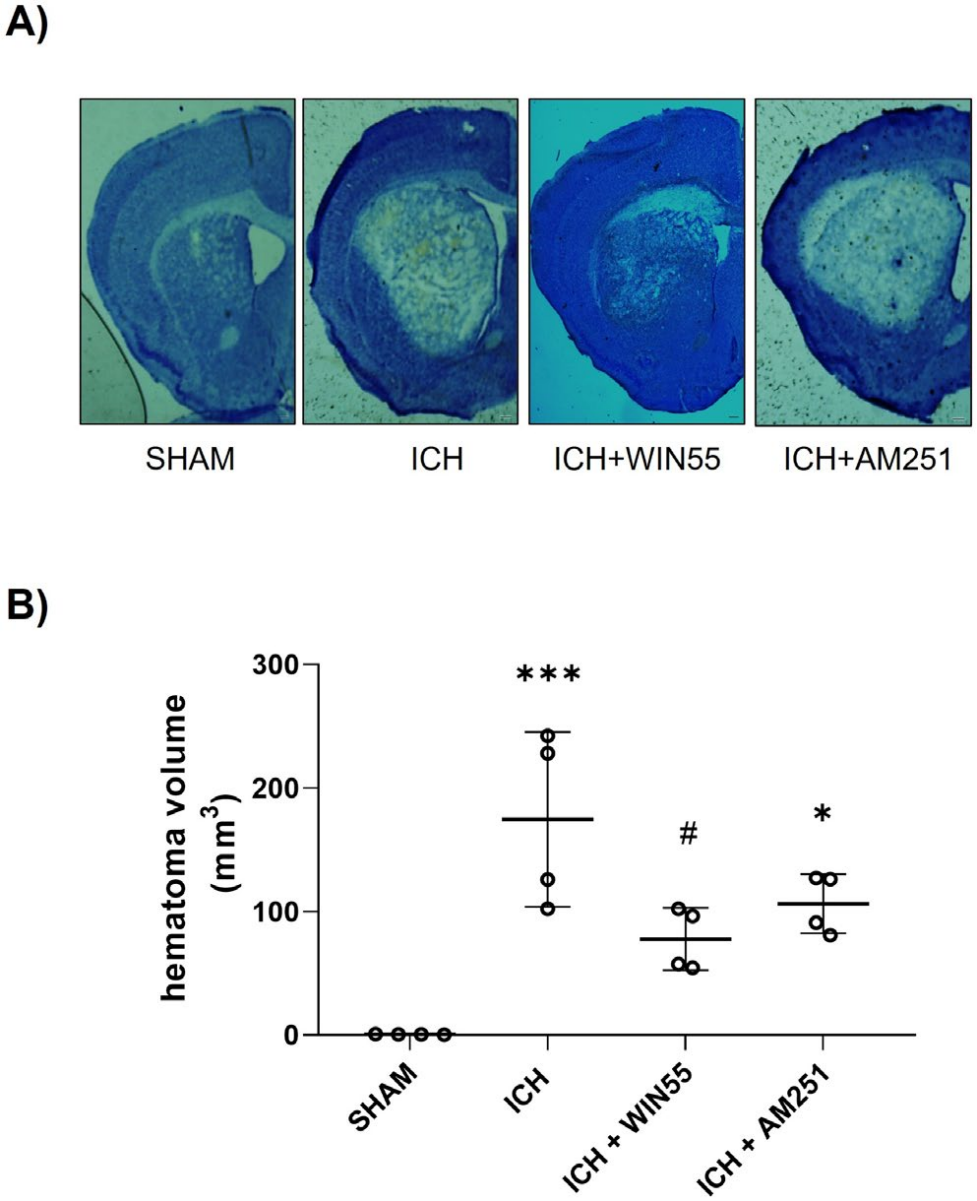
Hematoma volume in animals after ICH and treated with WIN55 or AM251. (A) Representative micrograph of coronal striatal sections stained with cresyl violet. The scale bars represent 200 μm. (B) Quantifying the hematoma volume is expressed in cubic millimeters (*n* = 4). Results were expressed as means ± standard deviation *p < 0.05, ***p < 0.001 vs. the SHAM; #*p* < 0.05 vs. the ICH group.

After ICH, mice in the cylinder test exhibited a significant increase in unilateral contacts (83.6% ± 17.6%) compared to the SHAM group (19.6% ± 9.2%). WIN55 treatment reduced these contacts to 36.1% ± 7.8%, while AM251 had no significant effect (74.6% ± 16.8%) (Figure 2A). In the inverted grid test, WIN55‐treated ICH mice had a holding time of 49.3 ± 12.7 s, significantly longer than those under ICH alone (11.6 ± 6 s) and those treated with AM251 (22.3 ± 12.4 s). SHAM mice held for 55 s (Figure 2B). Hematoma volume was evaluated post‐ICH as described in Materials and Methods. Figure 3 shows that the treated group has a larger lesion volume (174.710 ± 70.742 mm^3^) than the SHAM group (0.717 ± 0.176 mm^3^). WIN55 treatment significantly reduced the hematoma size induced by ICH to 77.767 ± 25.216 mm^3^, while AM251 did not alter the hematoma volume (106.38 ± 23.931 mm^3^), as depicted in Figure 3A,B.

### 3.3 WIN55 Reduces Brain Edema, Prevents BBB Disruption, and Lowers AQP4 Levels Following ICH

**FIGURE 4:**
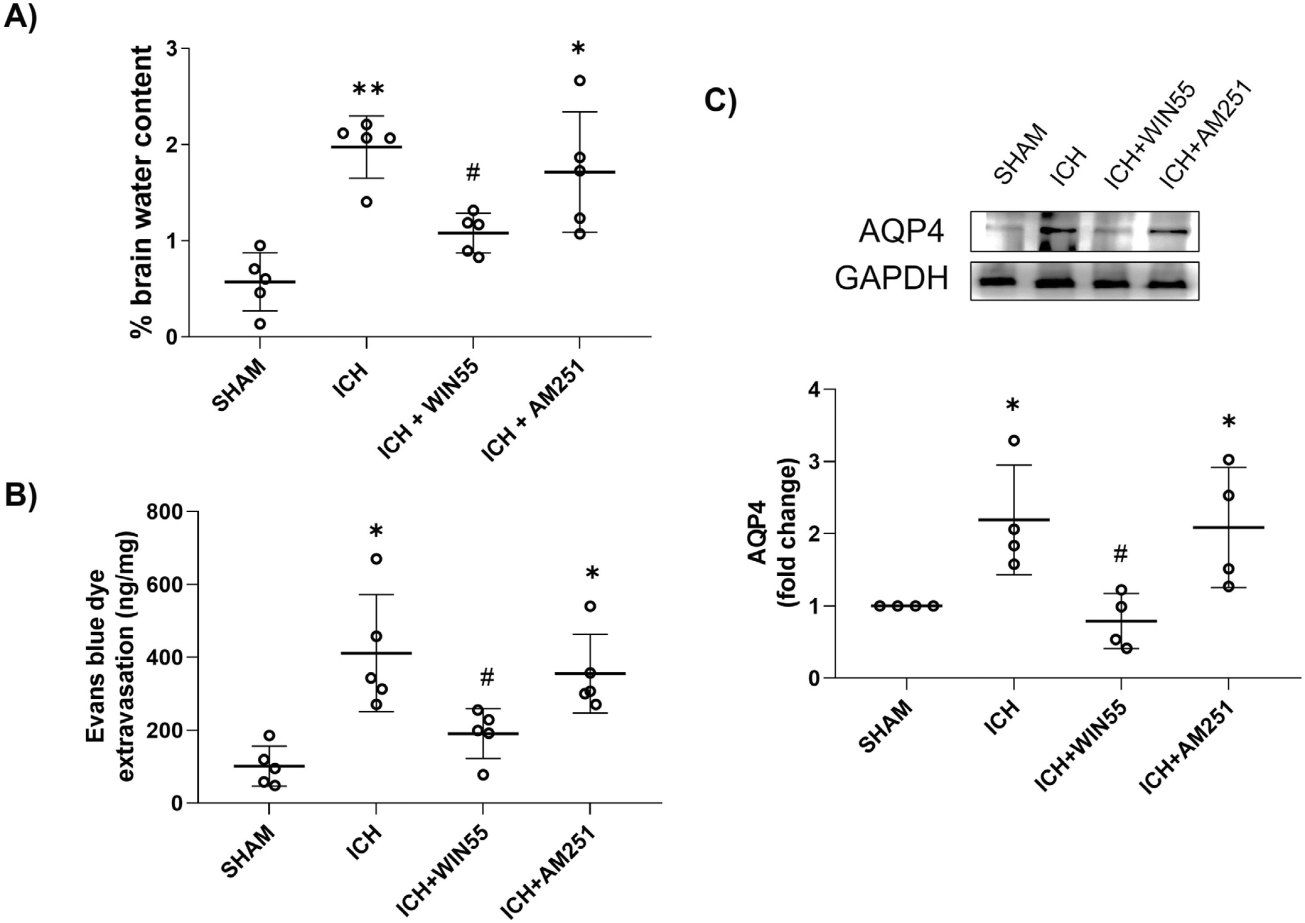
(A) Brain water content in animals after ICH and treated with WIN55 or AM251 (*n* = 5). (B) Evans Blue leakage quantification after ICH and treatments (*n* = 5). (C) AQP4 levels after ICH and treatments (*n* = 4). Representative western blot images and protein levels of AQP4 (39 kDa) GAPDH (37 kDa). Results are means ± standard deviation. *p < 0.05, **p < 0.01, vs. the SHAM group; #*p* < 0.05 vs. the ICH group.

After 24 h post‐injury, brain water content increased to 1.97% ± 0.32%, which was significantly higher than that observed in SHAM animals (0.57% ± 0.3%). This condition was reduced by more than 50% when animals were treated with WIN55 (1.07% ± 0.20%) (Figure 4A), while the AM251‐treated group subjected to ICH showed no significant change in water content (1.71% ± 0.62%) compared to ICH animals.

Evans blue leakage during ICH was significantly higher (411.20 ± 160.61 μg/mg) than in the SHAM group (101.58 ± 55.09 μg/mg), but WIN55 treatment markedly reduced BBB permeability (190.86 ± 67.78 μg/mg). In contrast, AM251 treatment (355.27 ± 108.03 μg/mg) did not significantly affect Evans blue leakage induced by ICH (Figure 4B). Additionally, ICH elevated AQP4 levels after 24 h, which were significantly reduced by treatment with WIN55 (Figure 4C), while treatment with AM251 did not affect AQP4 levels.

### 3.4 WIN‐55 Reduced the Increase in the NOX‐2 Catalytic Subunit gp91phox and NOX Activity Induced by ICH

**FIGURE 5:**
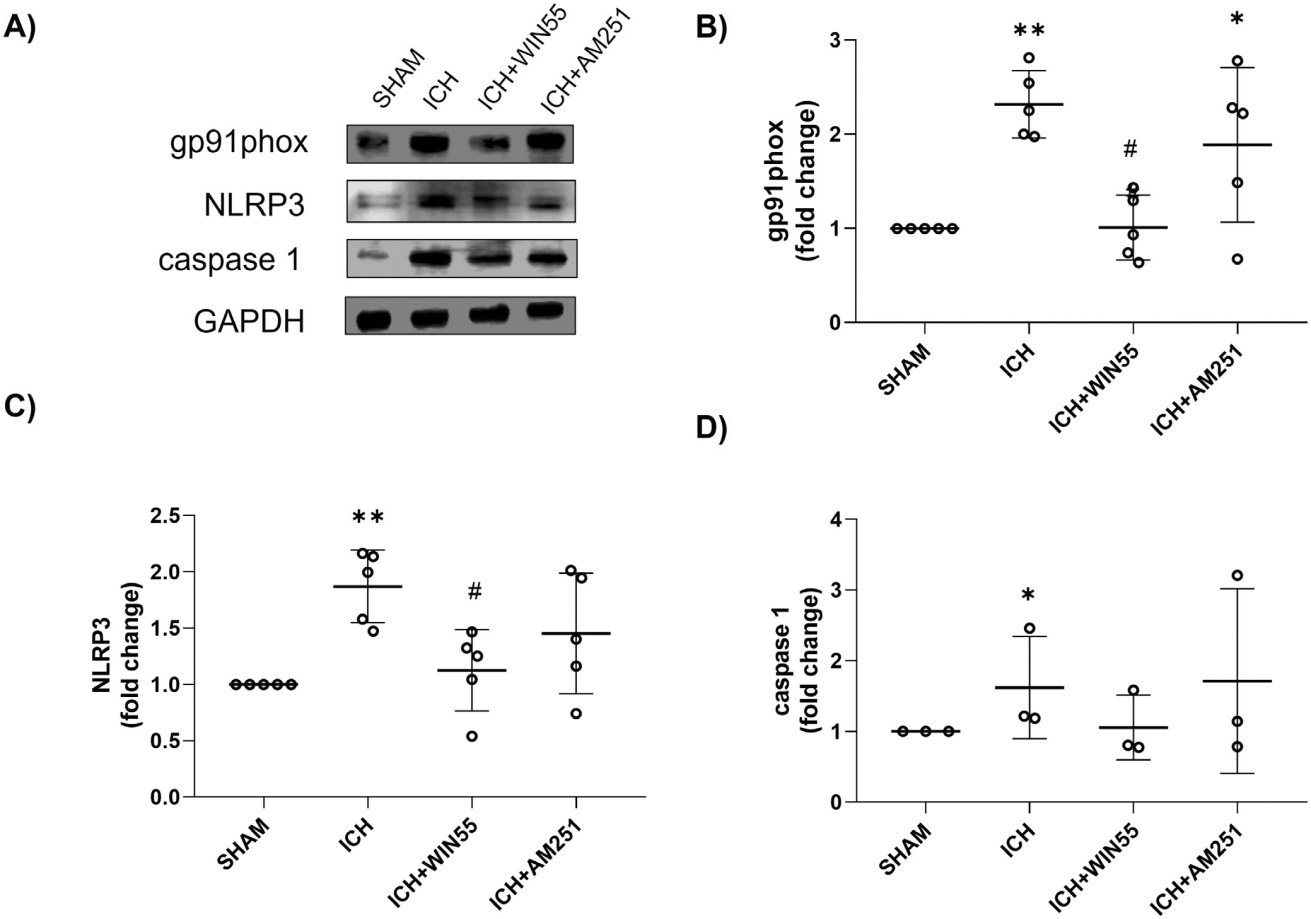
Protein levels of gp91phox, NLRP3, and caspase‐1 in animals after the ICH and treated with WIN55 or AM251. (A) Western Blot of gp91phox, NLRP3, caspase 1, and GAPDH representative images (*n* = 5). (B) Levels of gp91phox. (C) Levels of NLRP3. (D) Levels of caspase‐1. Results are expressed as fold change. Values represent the average ± standard deviation. gp91phox (37 KDa), NLRP3 (110 kDa). GAPDH (37 kDa), Caspase 1 (45 kDa). *p < 0.05, **p < 0.01, vs. the SHAM group; #*p* < 0.05 vs. the ICH group.

**FIGURE 6:**
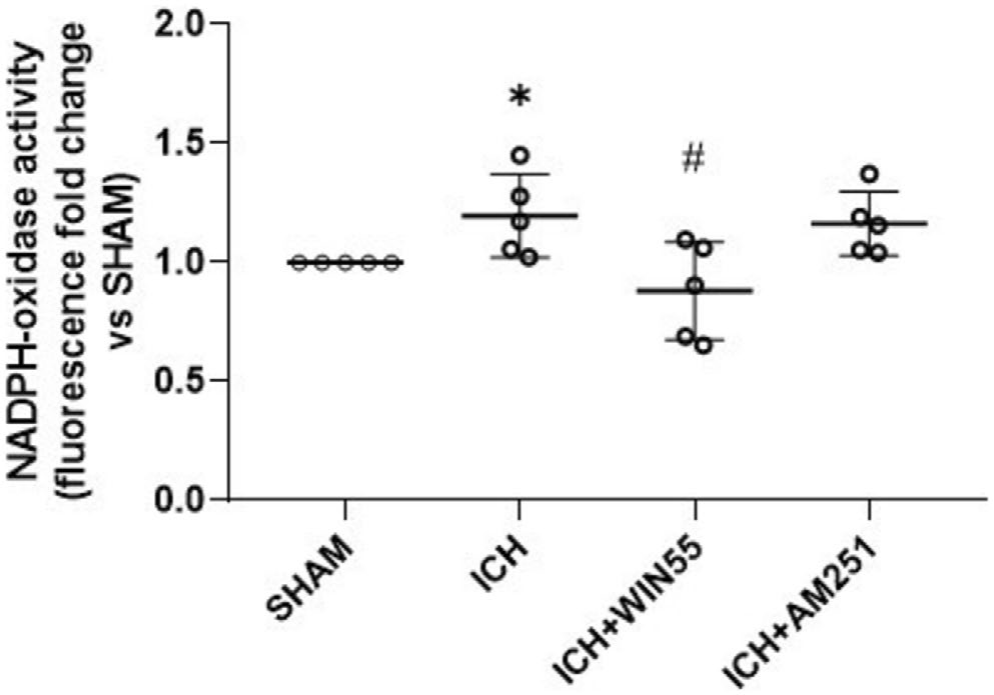
NOX activity in animals after the ICH and treated with WIN55 or AM251 (*n* = 5). Data are expressed as fold change of Et fluorescence relative to the SHAM group. Values were expressed as means ± standard deviation. *p < 0.05 vs. the corresponding SHAM group; #*p* < 0.05 vs. the ICH group.

As shown in Figure 5, gp91^phox^ protein levels were significantly elevated in the ICH group compared to the SHAM group. However, this increase was markedly diminished in animals treated with WIN55, while AM251 had no effect (Figure 5A,B). Striatal NOX activity also significantly increased 24 h post‐ICH, compared to the SHAM group (1.90 ± 0.46, 1.52 ± 0.09, and 1.75 ± 0.21‐fold, respectively), and this increase persisted with AM251 treatment (1.88 ± 0.32, 1.46 ± 0.09, and 1.88 ± 0.04‐fold). In contrast, the WIN55 treatment led to a significant decrease in enzyme activity (1.32 ± 0.56, 1.14 ± 0.24, and 0.92 ± 0.16‐fold) (Figure 6).

### 3.5 WIN55 Reduced NLRP3 Levels Induced by ICH

Regarding NLRP3, WIN55 significantly reduced protein levels compared to the high levels observed in the ICH group (Figure 5A,C). The caspase‐1 levels increased in the ICH group compared with the SHAM group. The level reduction was insignificant after the WIN55 treatment (Figure 5A,D).

### 3.6 MCC950 and WIN55 Attenuate Motor Dysfunction and Reduce the Hematoma Volume Induced by ICH

**FIGURE 7:**
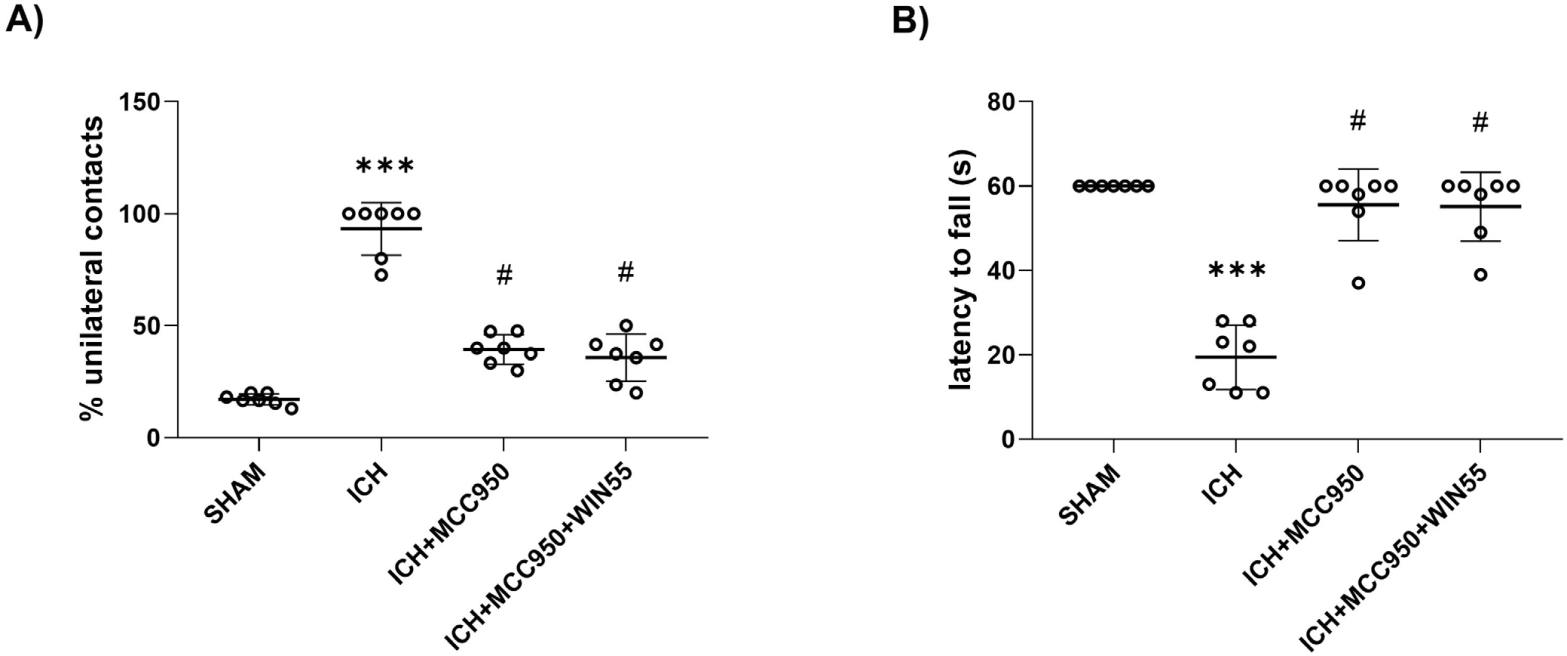
Motor activity recovery in animals after ICH treated with MCC950 or MCC950 plus WIN55. (A) Cylinder test. (B) Inverted grid test (*n* = 7). Results were expressed as means ± standard deviation. *p < 0.05, ***p < 0.001 vs. the SHAM group; #*p* < 0.05 vs. the ICH group.

In the cylinder test, ICH mice showed a higher percentage of unilateral contacts (93.2% ± 11.7%) compared to the SHAM group (17.1% ± 2.5%). Treatment with the NLRP3 inhibitor, MCC950, significantly reduced unilateral contacts to 39.4% ± 6.5%, while the combination of MCC950 and WIN55 resulted in a similar reduction (35.7% ± 10.5%) (Figure 7A). In the inverted grid test, both MCC950 and WIN55 treatments led to significantly longer holding times (55.5 ± 8.4 s and 55.1 ± 8.1 s, respectively) compared to the vehicle group (19.4 ± 7.6 s), with SHAM mice holding for 60 s (Figure 7B).

**FIGURE 8:**
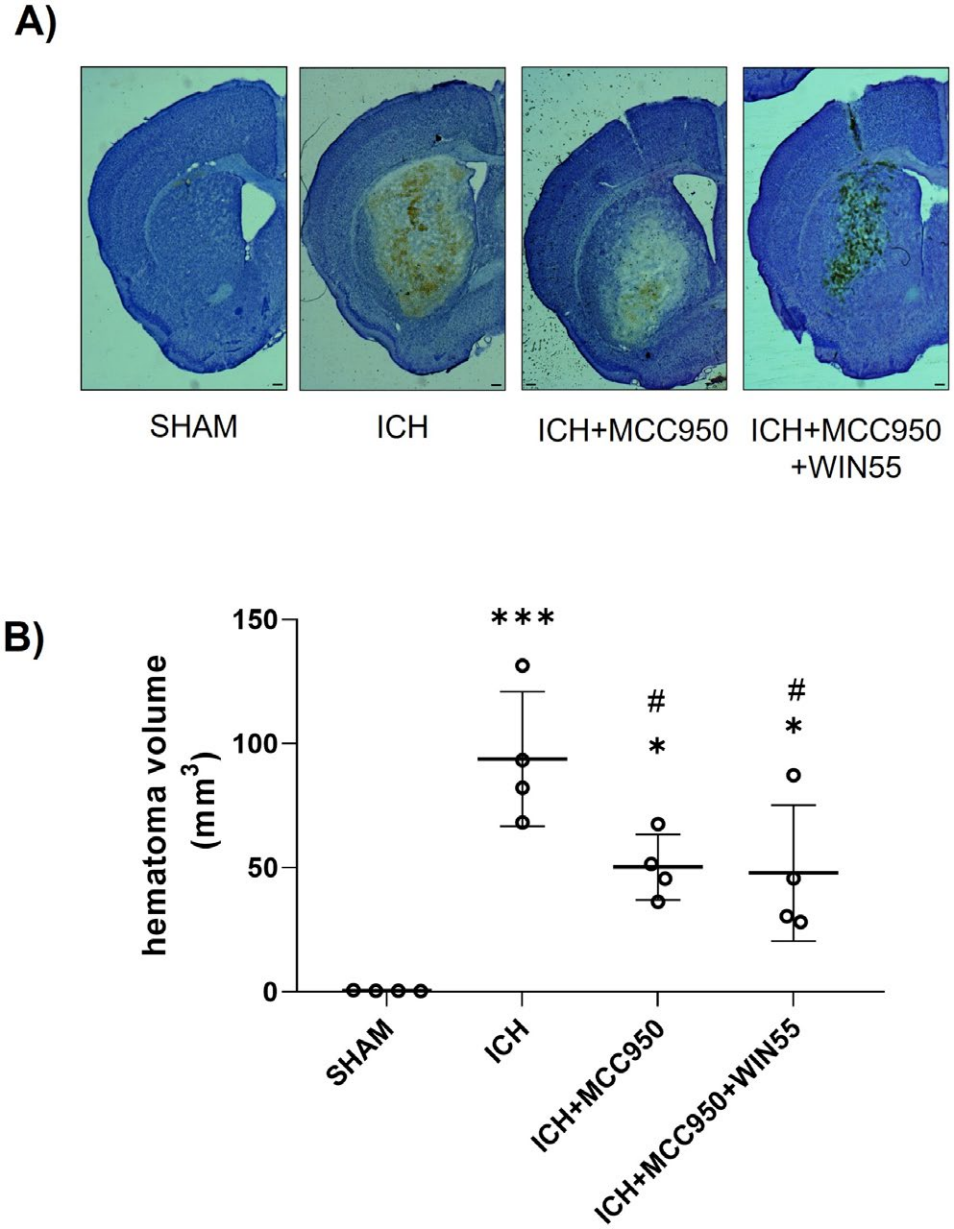
Hematoma volume in animals after ICH and treated with MCC950 or MCC950 plus WIN55. (A) Representative micrograph of coronal striatal sections stained with cresyl violet. The scale bars represent 200 μm. (B) Quantifying the hematoma volume is expressed in cubic millimeters (*n* = 4). Results were expressed as means ± standard deviation. *p < 0.05, ***p < 0.001 vs. the SHAM group; #*p* < 0.05 vs. the ICH group.

After the ICH, animals treated with MCC950 showed a reduction in hematoma volume (50.26 ± 13.19 mm^3^) compared to animals treated with vehicle (93.85 ± 27.13 mm^3^). The combination treatment with MCC950 and WIN55 exhibited a reduction in the hematoma size (47.91 ± 27.37 mm^3^), without statistical significance compared to animals treated with MCC950 alone (Figure 8A,B).

### 3.7 The Brain Edema Was Lower in Animals Treated With MCC950 and MCC950 Plus WIN55 MC

**FIGURE 9:**
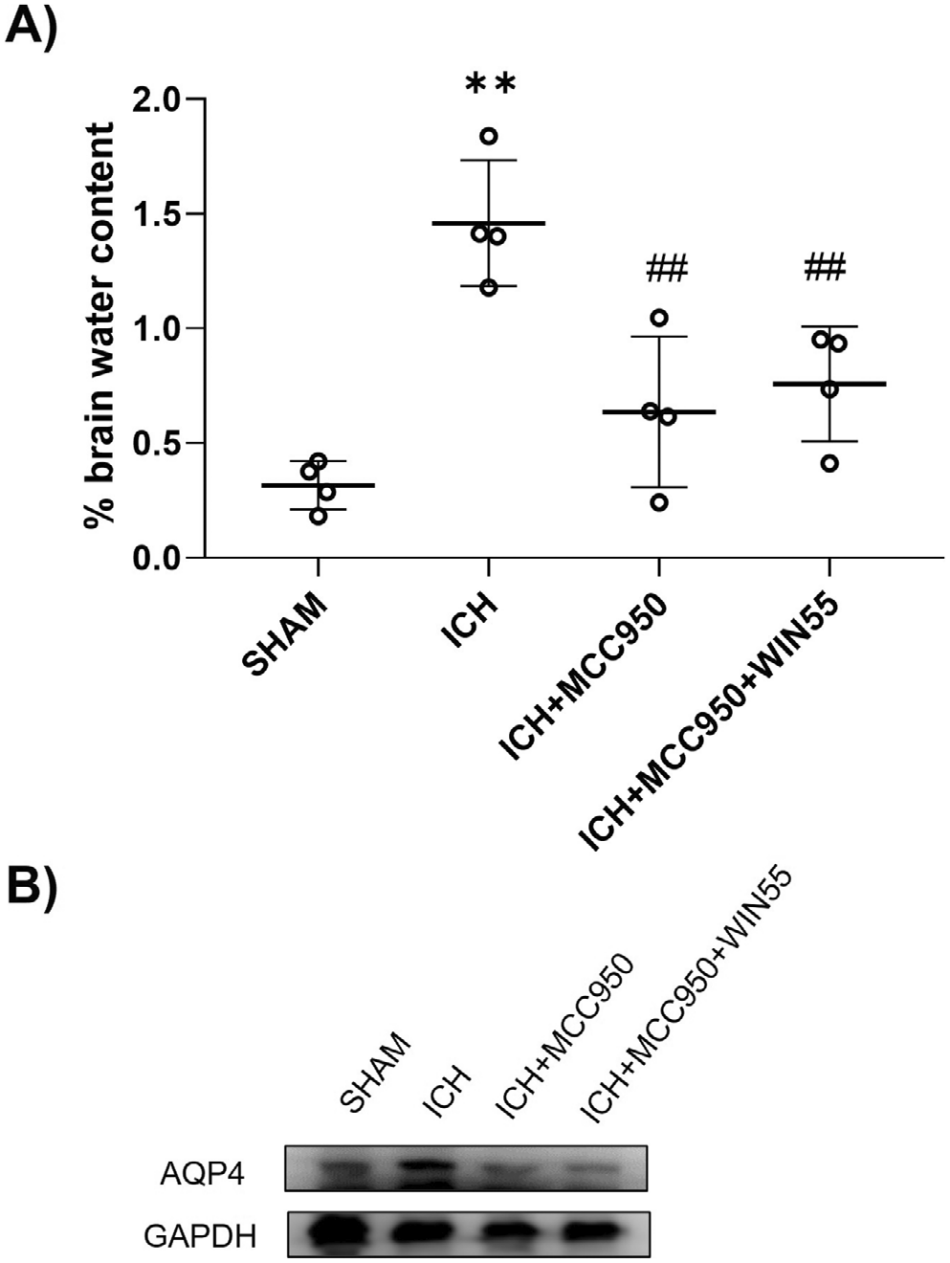
(A) Brain water content in animals after ICH treated with MCC950 or MCC950 plus WIN55 (*n* = 4). (B) Representative western blot image of AQP4 (39 kDa) and GAPDH (37 kDa). Results are means ± standard deviation. **p < 0.01, vs. the SHAM group; ##*p* < 0.01 vs. the ICH group.

After ICH, brain water content increased to 1.45% ± 0.27%, but treatment with MCC950 lowered it to 0.63% ± 0.32%. Similarly, the combination treatment of MCC950 plus WIN55 significantly reduced the brain water content (0.75% ± 0.25%) (Figure 9A). Both treatments also attenuated the increases in AQP4 levels observed within the first 24 h post‐ICH (Figure 9B).

## 4 Discussion

Oxidative stress and neuroinflammation following ICH contribute to edema progression, BBB disruption, and neuronal cell death. On the other hand, it is known that ECS activates neuromodulatory mechanisms in response to neuronal injury, with CB1 being the primary cannabinoid receptor expressed in neurons and participates in these modulatory mechanisms. In the present study, our observations indicate an increase in both cytosolic and membrane‐bound CB1 levels in response to ICH. This finding suggests an early activation of the ECS in neurons located in the periphery of the hematoma. An increase in CB1 levels has also been reported in animal models of stroke and chronic intermittent hypoxia [37, 38].

We also observed that ICH caused significant brain damage and motor dysfunction. Treatment with WIN55, a cannabinoid receptor agonist for CB1 and CB2 receptors, exerts neuroprotective effects in acquired brain injuries. Here, we found that the administration of WIN55 after ICH improved muscular strength and voluntary movement in the animals. Furthermore, motor dysfunction in mice treated with the CB1 antagonist AM251 suggests that the CB1 receptor plays a role in the neuroprotective mechanisms induced by WIN55.

A significant correlation was found between reduced hematoma volume in WIN55‐treated mice and improved sensorimotor function. Comparable results were observed with the CB1 agonist arachidonyl‐2‐chloroethylamide (ACEA), following brain ischemia–reperfusion injury [39].

The increase in AQP4 levels during ICH is associated with BBB rupture and brain edema. During ICH, these molecular processes are pivotal in the enlargement of hematomas and the elevation of intracranial pressure. WIN55 administration reduced both hematoma volume and brain water content, thereby mitigating hematoma expansion and motor impairment. It has been observed in a model of permanent cerebral ischemia that JZL‐184, an inhibitor of endocannabinoid degradation, reduced brain swelling, the area of infarction, and neurological impairment [40]. The disruption of the BBB contributes to the onset of the vasogenic phase of edema. In the present study, WIN55 treatment was shown to attenuate BBB damage, which corresponded with a decrease in edema formation. The protective effects of the ECS on the BBB have been previously reported with CBD, a dual agonist of CB1 and CB2 receptors, in an animal model of closed head injury [41]. Furthermore, in an alternative model of traumatic brain injury, the treatment with two endocannabinoid degradation inhibitors, JZL‐184 and URB597, was effective in reducing BBB disruption and neurological impairment [42].

The increases in AQP4 levels during the ICH have been previously reported [43, 44]. In the present study, WIN55 treatment significantly decreased AQP4 levels, correlating with reduced brain edema and disruption of the BBB. The upregulation of AQP4 at 3 and 7 days post‐ICH, was associated with decreased vasogenic edema and neuronal cell death, as it promotes the clearance of excess water accumulation [45]. In our study, AQP4 levels were assessed at 24 h post‐ICH; it is plausible that this time coincided with the cytotoxic phase of edema development. During the cytotoxic phase, the increase in AQP4 levels may contribute to astrocyte swelling and subsequent BBB disruption [46].

It has been shown that the NLRP3 inflammasome is activated after brain injury [18]. The activation of the NLRP3 inflammasome initiates the release of pro‐inflammatory cytokines, thereby exacerbating neuroinflammation associated with ICH [9]. Here, we found that following treatment with WIN55, NLRP3 levels were significantly reduced; however, the decrease in caspase 1 levels was not statistically significant. The ECS has been proposed to modulate inflammasome signaling, thereby regulating neuroinflammation [47]. In an animal model of Parkinson's disease, treatment with CBD reduced NLRP3 and caspase 1 levels, as well as improvements in neurological impairment [48]. Furthermore, the activation of CB2 receptor has been implicated in the NLRP3 inflammasome ubiquitination during spinal cord injury [49], reducing inflammation and neuronal death [23, 24]. WIN55 acts as an agonist for both CB1 and CB2 receptors. Nevertheless, the blockade of the CB1 receptor 30 min before ICH reverses the neuroprotective effects exerted by WIN55, suggesting an early response mediated by the CB1 receptor (Figure S1). Despite this result, the participation of the CB2 receptor in neuroprotection must be considered.

We observed decreased gp91phox levels in WIN55‐treated mice, which correlated with lower NOX activity. WIN55 treatment in a Parkinson's disease model reduced the translocation of NOX‐2 subunits Rac‐1 and p47phox to the plasma membrane [27]. Thus, the observed decrease of NOX activity induced by WIN55 suggests reduced ROS levels and indicates a close correlation between low NLRP3 levels and NOX‐2 involvement in inflammasome activation.

To elucidate the role of the inflammasome in the neuroprotective effects of WIN55, MCC950 was used to inhibit NLRP3 activation. The inhibition of the inflammasome before ICH reduced sensorimotor impairment, hematoma volume, and brain edema. Notably, the mice treated with WIN55 exhibited a similar improvement in the evaluated parameters. These results suggest that the neuroprotective effects of WIN55 could be mediated by the molecular pathways associated with inflammasome activation. In this context, it was demonstrated that the activation of the cAMP/PKA molecular pathway downregulates NLRP3 expression and attenuates neuroinflammation and neurological dysfunction [50]. It is also known that WIN55 stimulates cAMP accumulation and may activate the PKA signaling pathway, leading to the downregulation of NLRP3 expression [51]. Similarly, CBD has been shown to reduce the release of the inflammasome protein IL‐1β in microglial cultures challenged with LPS [52, 53]. In the same way, the transcription factor NF‐κB is activated in response to endogenous cytokines during the primal signal of the inflammasome activation, leading to the up‐regulation of NLRP3 and pro‐IL‐1β levels [54]. In an animal model of the excitotoxic lesion, activating the CB1 receptor with WIN55 inhibited the up‐regulation of the p65NF‐κB levels [55]. Similar results were reported during traumatic injury in mice after treatment with 2‐AG. This effect was not observed in CB1 receptor knockout mice, suggesting the regulation of NF‐κB by CB1 receptor after brain injury [56].

In conclusion, our results indicate that WIN55 exerts a neuroprotective effect during ICH by inhibiting NLRP3 activation via the regulation of NOX‐2 activity. Additional investigations are required to clarify the participation of the CB2 receptor in the WIN55‐mediated actions and to evaluate the role of the PKA and NF‐κB signaling pathways, which may elucidate the therapeutic potential of endocannabinoid receptors in hemorrhagic stroke.

## Author Contributions

A.M.‐T. conducted the experiments, participated in the design of the study, and contributed to the analysis and interpretation of the data, as well as to the writing of the manuscript. C.R.‐C. performed the WB and contributed to the analysis and interpretation of the data. J.M. participated in the design of the study, coordinated the study, raised funds, and contributed to the writing of the manuscript. All authors have read and approved the submitted version of the manuscript.

## Ethics Statement

This study was carried out following the accepted standards of animal care and with the procedures approved by the local Animal Care and Use Committee of the Instituto de Fisiología Celular, Universidad Nacional Autónoma de México (protocol number JMA222‐23). The protocol used followed the Guidelines for the Care and Use of Mammals in Neuroscience as well as guidelines released by the Mexican Institutes of Health Research and the National Institutes of Health Guide for the Care and Use of Laboratory Animals (NIH Publication No. 8023, revised 1978). All efforts were made to minimize animal suffering and to reduce the number of animals used.

## Conflicts of Interest

The authors declare no conflicts of interest.

## Supporting information


Appendix S1.

## Data Availability

The raw data supporting the conclusions of this manuscript will be made available by the authors, without undue reservation, to any qualified researcher.

## References

[1] V. Mazzoleni , A. Padovani , and A. Morotti , “Emergency Management of Intracerebral Hemorrhage,” Journal of Critical Care 74 (2023): 154232. 36565647 10.1016/j.jcrc.2022.154232

[2] D. A. Wilkinson , A. S. Pandey , B. G. Thompson , R. F. Keep , Y. Hua , and G. Xi , “Injury Mechanisms in Acute Intracerebral Hemorrhage,” Neuropharmacology 134 (2018): 240–248, 10.1016/j.neuropharm.2017.09.033. 28947377 PMC6027647

[3] Y. Wan , K. G. Holste , Y. Hua , R. F. Keep , and G. Xi , “Brain Edema Formation and Therapy After Intracerebral Hemorrhage,” Neurobiology of Disease 176 (2023): 105948, 10.1016/j.nbd.2022.105948. 36481437 PMC10013956

[4] Y. S. Feng , Z. X. Tan , L. Y. Wu , F. Dong , and F. Zhang , “The Involvement of NLRP3 Inflammasome in the Treatment of Alzheimer's Disease,” Ageing Research Reviews 64 (2020): 101192, 10.1016/j.arr.2020.101192. 33059089

[5] Y. Cao , Y. Wang , X. Li , X. Yang , B. Zeng , and Z. Guo , “MCC950 Ameliorates Cognitive Function by Reducing White Matter Microstructure Damage in Rats After SAH,” Brain Research Bulletin 202 (2023): 110743, 10.1016/j.brainresbull.2023.110743. 37591025

[6] S. Ismael , L. Zhao , S. Nasoohi , and T. Ishrat , “Inhibition of the NLRP3‐Inflammasome as a Potential Approach for Neuroprotection After Stroke,” Scientific Reports 8, no. 1 (2018): 5971, 10.1038/s41598-018-24350-x. 29654318 PMC5899150

[7] Y. Luo , J. Lu , W. Ruan , X. Guo , and S. Chen , “MCC950 Attenuated Early Brain Injury by Suppressing NLRP3 Inflammasome After Experimental SAH in Rats,” Brain Research Bulletin 146 (2019): 320–326, 10.1016/j.brainresbull.2019.01.027. 30716395

[8] Z. Zhang , P. Guo , S. Huang , et al., “Inhibiting Microglia‐Derived NLRP3 Alleviates Subependymal Edema and Cognitive Dysfunction in Posthemorrhagic Hydrocephalus After Intracerebral Hemorrhage via AMPK/Beclin‐1 Pathway,” Oxidative Medicine and Cellular Longevity 2022 (2022): 4177317, 10.1155/2022/4177317. 35620574 PMC9129981

[9] Q. Ma , S. Chen , Q. Hu , H. Feng , J. H. Zhang , and J. Tang , “NLRP3 Inflammasome Contributes to Inflammation After Intracerebral Hemorrhage,” Annals of Neurology 75, no. 2 (2014): 209–219, 10.1002/ana.24070. 24273204 PMC4386653

[10] K. Chen , B. Xu , X. Xiao , et al., “Involvement of CKS1B in the Anti‐Inflammatory Effects of Cannabidiol in Experimental Stroke Models,” Experimental Neurology 373 (2024): 114654, 10.1016/j.expneurol.2023.114654. 38104887

[11] M. Fang , F. Xia , J. Wang , et al., “The NLRP3 Inhibitor, OLT1177 Attenuates Brain Injury in Experimental Intracerebral Hemorrhage,” International Immunopharmacology 131 (2024): 111869, 10.1016/j.intimp.2024.111869. 38492343

[12] Y. Yingze , J. Zhihong , J. Tong , et al., “NOX2‐Mediated Reactive Oxygen Species Are Double‐Edged Swords in Focal Cerebral Ischemia in Mice,” Journal of Neuroinflammation 19, no. 1 (2022): 184, 10.1186/s12974-022-02551-6. 35836200 PMC9281066

[13] W. Wang , X. Zhang , L. Lin , J. Ren , R. He , and K. Sun , “Inhibition of NADPH Oxidase 2 (NOX2) Reverses Cognitive Deficits by Modulating Excitability and Excitatory Transmission in the Hippocampus After Traumatic Brain Injury,” Biochemical and Biophysical Research Communications 617 (2022): 1–7, 10.1016/j.bbrc.2022.05.002. 35660876

[14] D. R. Hernández‐Espinosa , L. Massieu , T. Montiel , and J. Morán , “Role of NADPH Oxidase‐2 in the Progression of the Inflammatory Response Secondary to Striatum Excitotoxic Damage,” Journal of Neuroinflammation 16, no. 1 (2019): 91, 10.1186/s12974-019-1478-4. 30995916 PMC6471795

[15] L. Gao , H. Shi , P. Sherchan , et al., “Inhibition of Lysophosphatidic Acid Receptor 1 Attenuates Neuroinflammation via PGE2/EP2/NOX2 Signalling and Improves the Outcome of Intracerebral Hemorrhage in Mice,” Brain, Behavior, and Immunity 91 (2021): 615–626, 10.1016/j.bbi.2020.09.032. 33035633 PMC13142233

[16] H. Deng , Y. Zhang , G. G. Li , et al., “P2X7 Receptor Activation Aggravates NADPH Oxidase 2‐Induced Oxidative Stress After Intracerebral Hemorrhage,” Neural Regeneration Research 16, no. 8 (2021): 1582–1591, 10.4103/1673-5374.303036. 33433488 PMC8323669

[17] L. Feng , Y. Chen , R. Ding , et al., “P2X7R Blockade Prevents NLRP3 Inflammasome Activation and Brain Injury in a Rat Model of Intracerebral Hemorrhage: Involvement of Peroxynitrite,” Journal of Neuroinflammation 12 (2015): 190, 10.1186/s12974-015-0409-2. 26475134 PMC4609067

[18] M. W. Ma , J. Wang , K. M. Dhandapani , and D. W. Brann , “NADPH Oxidase 2 Regulates NLRP3 Inflammasome Activation in the Brain After Traumatic Brain Injury,” Oxidative Medicine and Cellular Longevity 2017 (2017): 6057609, 10.1155/2017/6057609. 28785377 PMC5529650

[19] L. Cristino , T. Bisogno , and V. Di Marzo , “Cannabinoids and the Expanded Endocannabinoid System in Neurological Disorders,” Nature Reviews. Neurology 16, no. 1 (2020): 9–29, 10.1038/s41582-019-0284-z. 31831863

[20] H. C. Lu and K. Mackie , “Review of the Endocannabinoid System,” Biological Psychiatry: Cognitive Neuroscience and Neuroimaging 6, no. 6 (2021): 607–615, 10.1016/j.bpsc.2020.07.016. 32980261 PMC7855189

[21] A. M. Martínez‐Torres and J. Morán , “Aquaporin 4 and the Endocannabinoid System: A Potential Therapeutic Target in Brain Injury,” Experimental Brain Research 242, no. 9 (2024): 2041–2058, 10.1007/s00221-024-06896-7. 39043897 PMC11306651

[22] M. Fujii , P. Sherchan , P. R. Krafft , W. B. Rolland , Y. Soejima , and J. H. Zhang , “Cannabinoid Type 2 Receptor Stimulation Attenuates Brain Edema by Reducing Cerebral Leukocyte Infiltration Following Subarachnoid Hemorrhage in Rats,” Journal of the Neurological Sciences 342, no. 1–2 (2014): 101–106, 10.1016/j.jns.2014.04.034. 24819918 PMC4067767

[23] L. Li , D. Yun , Y. Zhang , et al., “A Cannabinoid Receptor 2 Agonist Reduces Blood‐Brain Barrier Damage via Induction of MKP‐1 After Intracerebral Hemorrhage in Rats,” Brain Research 1697 (2018): 113–123, 10.1016/j.brainres.2018.06.006. 29886251

[24] L. Lin , T. Yihao , F. Zhou , et al., “Inflammatory Regulation by Driving Microglial M2 Polarization: Neuroprotective Effects of Cannabinoid Receptor‐2 Activation in Intracerebral Hemorrhage,” Frontiers in Immunology 8 (2017): 112, 10.3389/fimmu.2017.00112. 28261199 PMC5306140

[25] E. Rangel‐López , A. L. Colín‐González , A. L. Paz‐Loyola , et al., “Cannabinoid Receptor Agonists Reduce the Short‐Term Mitochondrial Dysfunction and Oxidative Stress Linked to Excitotoxicity in the Rat Brain,” Neuroscience 285 (2015): 97–106, 10.1016/j.neuroscience.2014.11.016. 25446347

[26] D. P. Wang , K. Kang , J. Sun , Q. Lin , Q. L. Lv , and J. Hai , “URB597 and Andrographolide Improve Brain Microvascular Endothelial Cell Permeability and Apoptosis by Reducing Oxidative Stress and Inflammation Associated With Activation of Nrf2 Signaling in Oxygen‐Glucose Deprivation,” Oxidative Medicine and Cellular Longevity 2022 (2022): 4139330, 10.1155/2022/4139330. 35602108 PMC9119762

[27] Y. C. Chung , E. Bok , S. H. Huh , et al., “Cannabinoid Receptor Type 1 Protects Nigrostriatal Dopaminergic Neurons Against MPTP Neurotoxicity by Inhibiting Microglial Activation,” Journal of Immunology (Baltimore, MD: 1950) 187, no. 12 (2011): 6508–6517, 10.4049/jimmunol.1102435. 22079984

[28] M. Zille , T. D. Farr , R. F. Keep , C. Römer , G. Xi , and J. Boltze , “Novel Targets, Treatments, and Advanced Models for Intracerebral Haemorrhage,” Biomedicine 76 (2022): 103880, 10.1016/j.ebiom.2022.103880. PMC8850756 35158309

[29] P. R. Krafft , W. B. Rolland , K. Duris , et al., “Modeling Intracerebral Hemorrhage in Mice: Injection of Autologous Blood or Bacterial Collagenase,” Journal of Visualized Experiments 67 (2012): e4289, 10.3791/4289. PMC3490262 23023153

[30] A. A. Karan , Y. S. Spivak , E. M. Suleymanova , K. A. Gerasimov , A. P. Bolshakov , and L. V. Vinogradova , “Distant Neuroinflammation Acutely Induced by Focal Brain Injury and Its Control by Endocannabinoid System,” Experimental Neurology 373 (2024): 114679, 10.1016/j.expneurol.2024.114679. 38190933

[31] L. Wang , Y. Zeng , Y. Zhou , et al., “Win55,212–2 Improves Neural Injury Induced by HIV‐1 Glycoprotein 120 in Rats by Exciting CB2R,” Brain Research Bulletin 182 (2022): 67–79, 10.1016/j.brainresbull.2022.02.006. 35157986

[32] M. Bellut , M. Bieber , P. Kraft , A. N. R. Weber , G. Stoll , and M. K. Schuhmann , “Delayed NLRP3 Inflammasome Inhibition Ameliorates Subacute Stroke Progression in Mice,” Journal of Neuroinflammation 20, no. 1 (2023): 4, 10.1186/s12974-022-02674-w. 36600259 PMC9811791

[33] K. L. Schaar , M. M. Brenneman , and S. I. Savitz , “Functional Assessments in the Rodent Stroke Model,” Experimental & Translational Stroke Medicine 2, no. 1 (2010): 13, 10.1186/2040-7378-2-13. 20642841 PMC2915950

[34] R. M. Deacon , “Measuring the Strength of Mice,” Journal of Visualized Experiments: JoVE 76 (2013): 2610, 10.3791/2610. PMC3725666 23770643

[35] P. Kozler , D. Marešová , and J. Pokorný , “Determination of Brain Water Content by Dry/Wet Weight Measurement for the Detection of Experimental Brain Edema,” Physiological Research 71, no. S2 (2022): S277–S283, 10.33549/physiolres.934996. 36647915 PMC9906661

[36] B. Ahishali and M. Kaya , “Evaluation of Blood‐Brain Barrier Integrity Using Vascular Permeability Markers: Evans Blue, Sodium Fluorescein, Albumin‐Alexa Fluor Conjugates, and Horseradish Peroxidase,” Methods in Molecular Biology (Clifton, NJ) 2367 (2021): 87–103, 10.1007/7651_2020_316. 32785841

[37] X. Gao , S. Wu , Y. Dong , et al., “Role of the Endogenous Cannabinoid Receptor 1 in Brain Injury Induced by Chronic Intermittent Hypoxia in Rats,” International Journal of Neuroscience 128, no. 9 (2018): 797–804, 10.1080/00207454.2017.1420069. 29264962

[38] K. L. Jin , X. O. Mao , P. C. Goldsmith , and D. A. Greenberg , “CB1 Cannabinoid Receptor Induction in Experimental Stroke,” Annals of Neurology 48, no. 2 (2000): 257–261. 10939579

[39] S. Yang , B. Hu , Z. Wang , et al., “Cannabinoid CB1 Receptor Agonist ACEA Alleviates Brain Ischemia/Reperfusion Injury via CB1‐Drp1 Pathway,” Cell Death Discovery 6 (2020): 102, 10.1038/s41420-020-00338-3. 33083022 PMC7548964

[40] M. R. Rahmani , A. Shamsizadeh , A. Moghadam‐Ahmadi , G. Bazmandegan , and M. Allahtavakoli , “JZL184, as a Monoacylglycerol Lipase Inhibitor, Down‐Regulates Inflammation in a Cannabinoid Pathway Dependent Manner,” Biomedicine & Pharmacotherapy = Biomedecine & Pharmacotherapie 103 (2018): 1720–1726, 10.1016/j.biopha.2018.05.001. 29864962

[41] H. Jiang , H. Li , Y. Cao , et al., “Effects of Cannabinoid (CBD) on Blood Brain Barrier Permeability After Brain Injury in Rats,” Brain Research 1768 (2021): 147586, 10.1016/j.brainres.2021.147586. 34289379

[42] P. S. Katz , J. K. Sulzer , R. A. Impastato , S. X. Teng , E. K. Rogers , and P. E. Molina , “Endocannabinoid Degradation Inhibition Improves Neurobehavioral Function, Blood‐Brain Barrier Integrity, and Neuroinflammation Following Mild Traumatic Brain Injury,” Journal of Neurotrauma 32, no. 5 (2015): 297–306, 10.1089/neu.2014.3508. 25166905 PMC4348366

[43] X. Liu , G. Wu , N. Tang , et al., “Glymphatic Drainage Blocking Aggravates Brain Edema, Neuroinflammation via Modulating TNF‐α, IL‐10, and AQP4 After Intracerebral Hemorrhage in Rats,” Frontiers in Cellular Neuroscience 15 (2021): 784154, 10.3389/fncel.2021.784154. 34975411 PMC8718698

[44] Z. Wang , Y. Li , Z. Wang , et al., “Edaravone Maintains AQP4 Polarity via OS/MMP9/β‐DG Pathway in an Experimental Intracerebral Hemorrhage Mouse Model,” Molecular Neurobiology 61, no. 10 (2024): 7639–7658, 10.1007/s12035-024-04028-4. 38421470

[45] H. Jeon , M. Kim , W. Park , et al., “Upregulation of AQP4 Improves Blood‐Brain Barrier Integrity and Perihematomal Edema Following Intracerebral Hemorrhage,” Neurotherapeutics 18, no. 4 (2021): 2692–2706, 10.1007/s13311-021-01126-2. 34545550 PMC8804112

[46] W. Han , Y. Song , M. Rocha , and Y. Shi , “Ischemic Brain Edema: Emerging Cellular Mechanisms and Therapeutic Approaches,” Neurobiology of Disease 178 (2023): 106029, 10.1016/j.nbd.2023.106029. 36736599

[47] S. V. Suryavanshi , I. Kovalchuk , and O. Kovalchuk , “Cannabinoids as Key Regulators of Inflammasome Signaling: A Current Perspective,” Frontiers in Immunology 11 (2021): 613613, 10.3389/fimmu.2020.613613. 33584697 PMC7876066

[48] L. Wang , X. Wu , G. Yang , et al., “Cannabidiol Alleviates the Damage to Dopaminergic Neurons in 1‐Methyl‐4‐Phenyl‐1,2,3,6‐Tetrahydropyridine‐Induced Parkinson's Disease Mice via Regulating Neuronal Apoptosis and Neuroinflammation,” Neuroscience 498 (2022): 64–72, 10.1016/j.neuroscience.2022.06.036. 35792194

[49] F. Jiang , M. Xia , Y. Zhang , et al., “Cannabinoid Receptor‐2 Attenuates Neuroinflammation by Promoting Autophagy‐Mediated Degradation of the NLRP3 Inflammasome Post Spinal Cord Injury,” Frontiers in Immunology 13 (2022): 993168, 10.3389/fimmu.2022.993168. 36238284 PMC9553321

[50] J. Huang , D. Tang , Y. Cao , et al., “Inhibition of PDE10A‐Rescued TBI‐Induced Neuroinflammation and Apoptosis Through the cAMP/PKA/NLRP3 Pathway,” Evidence‐Based Complementary and Alternative Medicine: Ecam 2022 (2022): 3311250, 10.1155/2022/3311250. 35463083 PMC9019408

[51] K. Eldeeb , S. Leone‐Kabler , and A. C. Howlett , “CB1 Cannabinoid Receptor‐Mediated Increases in Cyclic AMP Accumulation Are Correlated With Reduced Gi/o Function,” Journal of Basic and Clinical Physiology and Pharmacology 27, no. 3 (2016): 311–322, 10.1515/jbcpp-2015-0096. 27089415 PMC5497837

[52] N. Rimmerman , A. Juknat , E. Kozela , R. Levy , H. B. Bradshaw , and Z. Vogel , “The Non‐Psychoactive Plant Cannabinoid, Cannabidiol Affects Cholesterol Metabolism‐Related Genes in Microglial Cells,” Cellular and Molecular Neurobiology 31, no. 6 (2011): 921–930, 10.1007/s10571-011-9692-3. 21533611 PMC11498456

[53] M. Dos‐Santos‐Pereira , F. S. Guimarães , E. Del‐Bel , R. Raisman‐Vozari , and P. P. Michel , “Cannabidiol Prevents LPS‐Induced Microglial Inflammation by Inhibiting ROS/NF‐κB‐Dependent Signaling and Glucose Consumption,” Glia 68, no. 3 (2020): 561–573, 10.1002/glia.23738. 31647138

[54] N. Kelley , D. Jeltema , Y. Duan , and Y. He , “The NLRP3 Inflammasome: An Overview of Mechanisms of Activation and Regulation,” International Journal of Molecular Sciences 20, no. 13 (2019): 3328, 10.3390/ijms20133328. 31284572 PMC6651423

[55] A. M. Martínez‐Torres and J. Morán , “CB1 Receptor Activation Provides Neuroprotection in an Animal Model of Glutamate‐Induced Excitotoxicity Through a Reduction of NOX‐2 Activity and Oxidative Stress,” CNS Neuroscience & Therapeutics 30, no. 11 (2024): e70099, 10.1111/cns.70099. 39496572 PMC11534500

[56] D. Panikashvili , R. Mechoulam , S. M. Beni , A. Alexandrovich , and E. Shohami , “CB1 Cannabinoid Receptors Are Involved in Neuroprotection via NF‐Kappa B Inhibition,” Journal of Cerebral Blood Flow and Metabolism: Official Journal of the International Society of Cerebral Blood Flow and Metabolism 25, no. 4 (2005): 477–484, 10.1038/sj.jcbfm.9600047. 15729296

