# Supplementary material for: Cannabinoid Receptors Reduced Early Brain Damage by Regulating NOX‐2 and the NLRP3 Inflammasome in an Animal Model of Intracerebral Hemorrhage
Source: CNS Neurosci Ther. 2025 Apr 17;31(4):e70385. doi: 10.1111/cns.70385 (PMC12005396; doi:10.1111/cns.70385)
Supplement: Supplementary file 1 — Appendix S1. [file CNS-31-e70385-s001.pdf]

Supplemental files

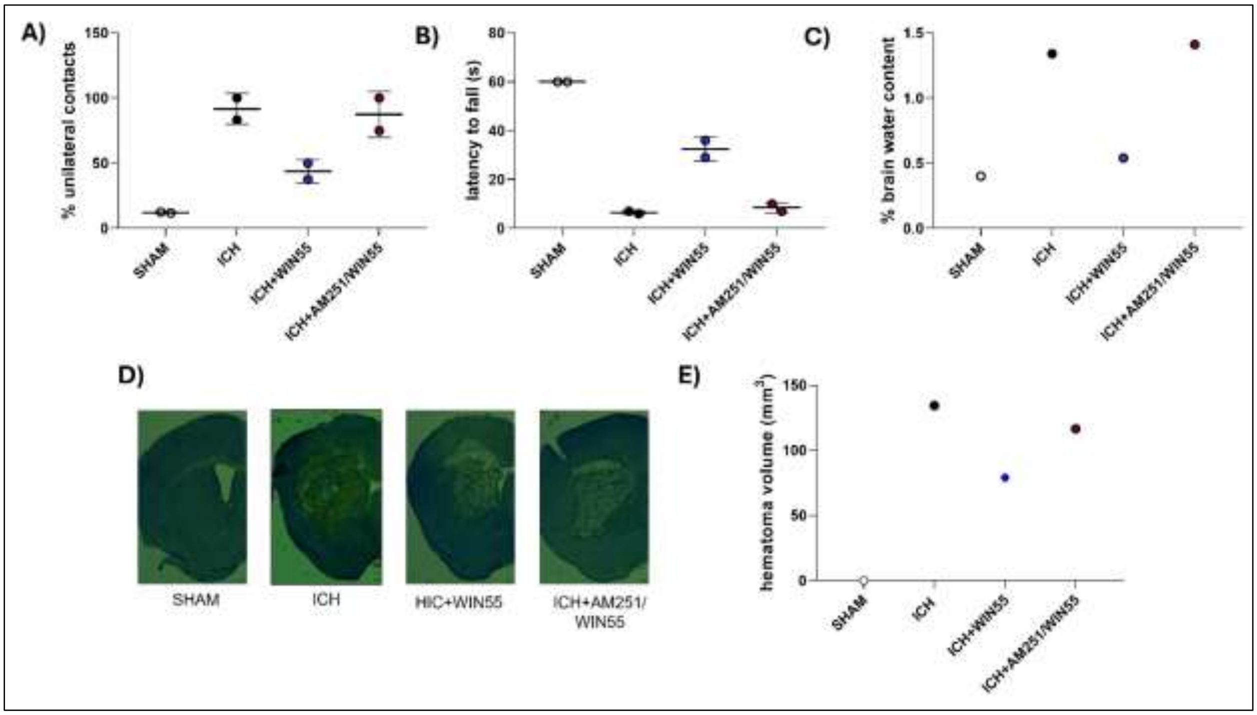

Figure 1. Motor activity recovery in animals after ICH and treated with WIN55 or AM251/WIN55. A Cylinder test. B Inverted grid test. C Brain water content in animals after ICH and treated with WIN55 or AM251/WIN55. Hematoma volume in animals after ICH and treated with WIN55 or AM251/WIN55 D Representative micrographs of coronal striatal sections stained with cresyl violet. The scale bars represent 200 μm. E Quantifying the hematoma volume is expressed in cubic millimeters.

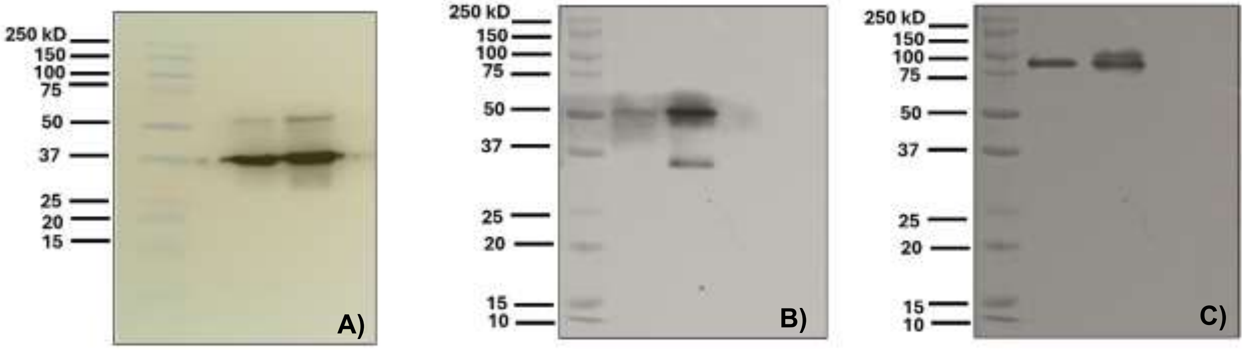

Full unedited blot for Figure 1. A) CB1 and GAPDH cytosolic fraction. B) CB1 membrane fraction. C) Na+/K+-ATPase α1 membrane fraction

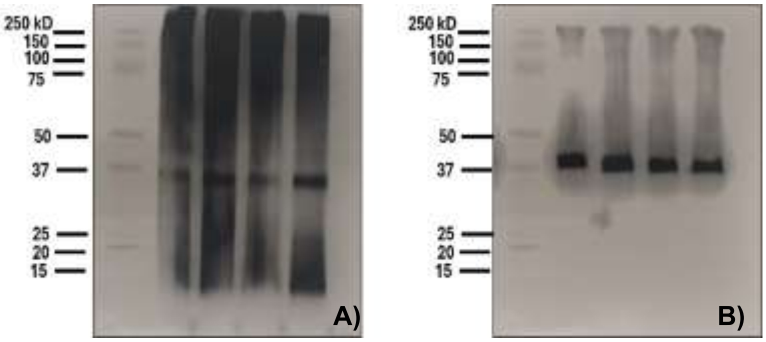

Full unedited blot for Figure 4. A) AQP4 B) GAPDH

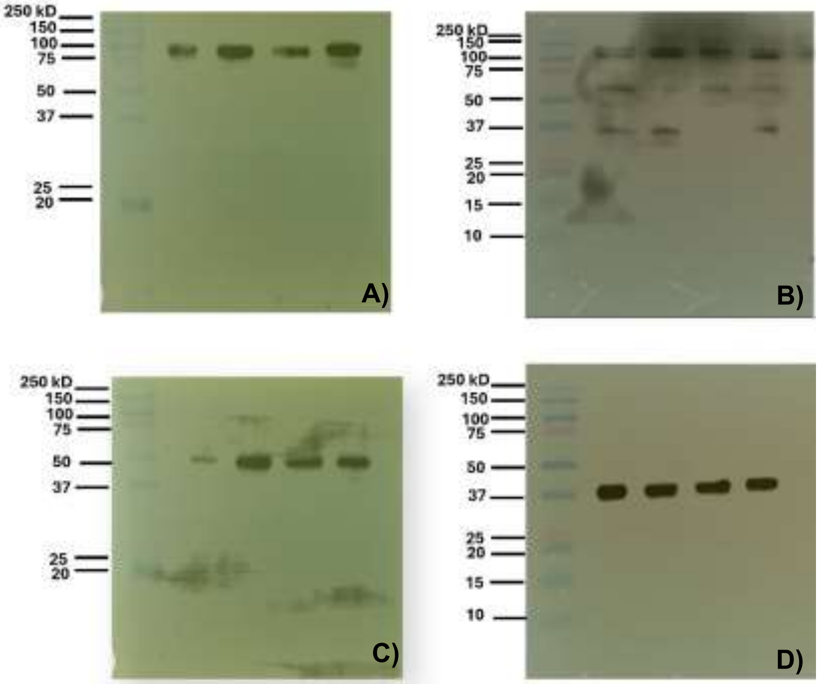

Full unedited blot for Figure 5. A) gp91phox B) NLRP3
C) caspase 1 D) GAPDH

D)

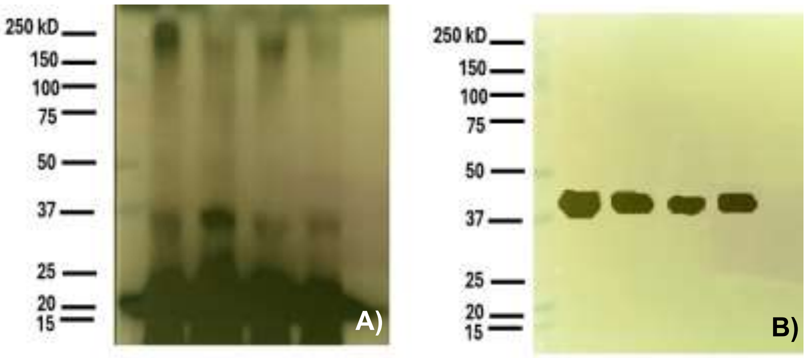

Full unedited blot for Figure 9. A) AQP4 B) GAPDH
